# Neurogenic effect of exercise via the thioredoxin-1/ extracellular regulated kinase/β-catenin signaling pathway mediated by β2-adrenergic receptors in chronically stressed dentate gyrus

**DOI:** 10.20463/jenb.2019.0018

**Published:** 2019-09-30

**Authors:** Mun-Hee Kim, Yea-Hyun Leem

**Affiliations:** 1. Department of Physical Education, Korea National Sport University, Seoul Republic of Korea; 2. Department of Molecular Medicine and Tissue Injury Defense Research Center, Ewha Womans University, Seoul Republic of Korea

**Keywords:** repeated stress, exercise, neurogenesis, β2-adrenergic receptor, thioredoxin-1

## Abstract

**[Purpose]:**

Chronic stress is a precipitating factor for depression, whereas exercise is beneficial for both the mood and cognitive process. The current study demonstrates the anti-depressive effects of regular exercise and the mechanisms linked to hippocampal neurogenesis.

**[Methods]:**

Mice were subjected to 14 consecutive days of restraint, followed by 3 weeks of treadmill running, and were then subjected to behavioral tests that included the forced swimming and Y-maze tests. Protein levels were assessed using western blot analysis and newborn cells were detected using 5-bromo-2′-deoxyuridine (BrdU).

**[Results]:**

Three weeks of treadmill running ameliorated the behavioral depression caused by 14 days of continuous restraint stress. The exercise regimen enhanced BrdU-labeled cells and class III β-tubulin levels in the hippocampal dentate gyrus, as well as those of thioredoxin-1 (TRX-1) and synaptosomal β2-adrenergic receptors (β2-AR) under stress. In vitro experiments involving treatment with recombinant human TRX-1 (rhTRX-1) augmented the levels of phospho-extracellular signal-regulated kinases 1 and 2 (ERK1/2), nuclear β-catenin, and proliferating cell nuclear antigens, which were previously inhibited by U0216 and FH535 (inhibitors of ERK1/2 and β-catenin/T cell factor-mediated transcription, respectively). The hippocampal neurogenesis elicited by a 7-day exercise regimen was abolished by a selective inhibitor of β2-AR, butoxamine.

**[Conclusion]:**

These results suggest that TRX-1-mediated hippocampal neurogenesis by β2-AR function is a potential mechanism underlying the psychotropic effect of exercise.

## INTRODUCTION

Stress leads to widespread alterations in the neurobiological and behavioral responses of the brain. Chronic stress is a precipitating factor in the etiogenesis of cognitive and mood abnormalities^1^^,^^2^.

The hippocampus is vulnerable to stress, especially the dentate gyrus (DG), which receives the most noradrenergic inputs via the β-adrenergic receptors (β-AR, β1-AR and β2-AR) from the locus coeruleus when compared to the cornu ammonis subfields CA1 and CA3^3^^,^^4^. This suggests that the DG is highly sensitive to noradrenergic inputs. Most of the evidence has demonstrated a neuromodulatory role of the central noradrenergic system in hippocampal function. For example, neuronal excitability is enhanced by noradrenaline (NA)-mediated β-AR signaling in the DG, in the CA subfields^3^^,^^5^. In terms of hippocampal neurogenesis, several studies have shown the neurogenic effect of NA, evidenced by NA-mediated activation of hippocampal precursors through β3-AR^6^. Moreover, NA transporter inhibitors such as desipramine and reboxetine have an anti-depressive effect, enhancing NA levels in the brain^7^. As mentioned before, β-AR signaling in the DG may be associated with chronic stress-induced mood and cognitive abnormalities. 

Thioredoxin-1 (TRX-1) is a 12 kDa oxidoreductase enzyme with two redox-active cysteine residues in its active site sequence: -Cys-Gly-Pro-Cys-. This molecule has anti-inflammatory, anti-apoptotic, and anti-oxidative properties against various environmental stressors, such as ultraviolet irradiation, H_2_O_2_, X-rays, and ischemic reperfusion. These activities happen via the thiol-disulfide exchange reaction, nuclear factor E2-related factor 2/antioxidant response element (Nrf2/ARE) signaling, and the apoptosis signal-regulating kinase 1/c-jun N-terminal kinase/p38 (ASK1/JNK/p38) pathway^8^^-^^10^. Furthermore, TRX-1 elicits a pleiotropic cellular effect of cell proliferation in tumor, endothelial, and adipose tissue-derived mesenchymal stem cells^11^^,^^12^. One study has demonstrated that the administration of recombinant human TRX-1 (rhTRX-1) increased the number of neural stem cells in mice subjected to bilateral common carotid artery occlusion^13^. Furthermore, TRX-1 expression was demonstrated to be regulated by β-AR signaling^14^^,^^15^. These studies suggest a probable hypothesis that the neurogenic effect of TRX-1 may be associated with β-AR signaling under stress conditions. 

Cumulative evidences have described the psychotropic activity of exercise^16^^,^^17^. Several studies have particularly demonstrated that the psychotropic effects of exercise were strongly related to hippocampal neurogenesis^18^^-^^20^. The neurogenic propensity of the hippocampus is recognized as a biological indicator of anti-depressive and stress responses^20^^,^^21^. Furthermore, exercise can enhance NA release and noradrenergic activity in many brain structures, including the hippocampus^22^^,^^23^. 

The beneficial effects of exercise on brain function, such as hippocampal neurogenesis, have been recognized. Exercise also facilitates NA neurotransmission in the brain. However, the mechanism underlying the psychotropic activity of exercise, which is associated to the relationship between hippocampal neurogenesis and β-AR-mediated TRX-1 signaling, has rarely been studied until now. Accordingly, we explored whether exercise contributed to β-AR-mediated cell proliferation through induction with TRX-1 under chronic stress conditions, focusing on the ERK/β-catenin signaling pathway.

## METHODS

### Mice

Seven-week-old C57BL/6 male mice were obtained from Daehan Biolink, Inc. (Eumsung, Chungbuk, Korea) and housed in clear plastic cages under specific pathogen-free conditions with a 12:12-h (light-dark) cycle. All of the mice had free access to standard irradiated chow (Purina Mills, Seoul, Korea). All of the experimental procedures used in this study were approved by the Institutional Animal Care and Use Committee at Ewha Womans University, based on the National Institutes of Health Guide for the Care and Use of Laboratory Animals.

### Experimental design

**Figure 1.**
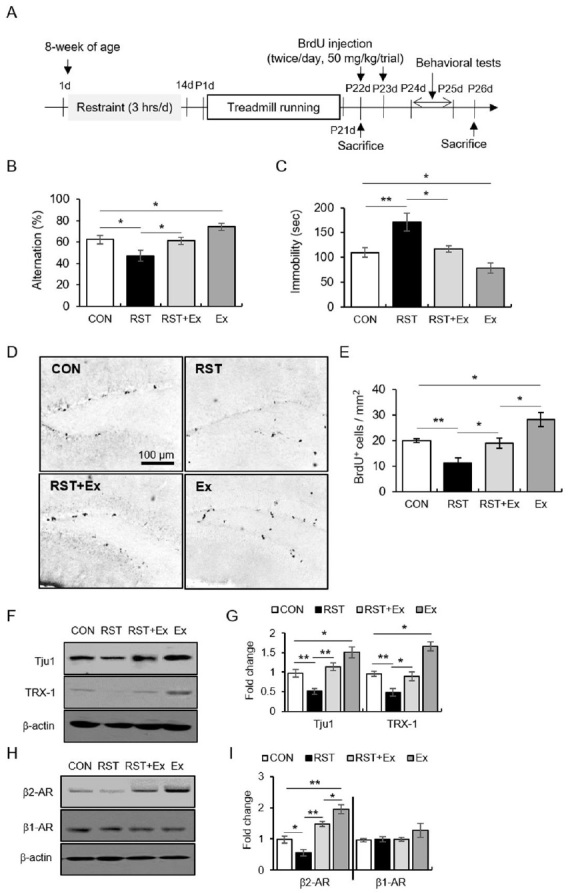
The anti-depressive effect of regular exercise, along with the altered hippocampal neurogenesis, TRX-1, and synaptosomal β2-AR levels after repeated restraint stress. (A) The experimental procedure. (B) Quantitative analysis of alternation in the Y-maze test. (C) Quantitative analysis of immobility in the FST. (D) Representative images of BrdU-labeled cells. (E) Quantitative analysis of BrdU-labeled cells. (F) Representative images of western blot data for Tju1 and TRX-1. (G) Quantitative analysis of Tju1 and TRX-1. (H) Representative images of western blot data of synaptic β2- and β1-AR. (I) Quantitative analysis of synaptic β2- and β1-AR. Data are presented as the mean ± standard error of the mean. *p < 0.05, **p < 0.01. RST (restraint stress treatment): chronic restraint stress; Ex: treadmill exercise; FST: forced swimming test.

For experiment 1 (Fig. 1), mice were divided into four groups (control: CON, restraint stress: RST, exercise combined with restraint stress: RST+Ex, and exercise: Ex). The procedures of chronic stress and exercise were described in our previous publication ^24^^,^^25^. The treadmill speed was gradually increased so as not to increase stress in the experimental animals (pre-exercise: 12 m/min, 20 min/day, 3 days; main exercise: 17 m/min, 60 min/day, 6 days/week). Subsequently, the mice underwent behavioral tests (forced swim test, FST; Y-maze test; N = 10 for each group) one day after the last exercise regimen (the FST test was performed one day after the Y-maze test). All mice were injected intraperitoneally with 5-bromo-2′-deoxyuridine (BrdU, Sigma-Aldrich, St. Louis, MO, USA; 50 mg/kg, twice per day, 2 h interval) during 2 days and were sacrificed 3 days later. Subjects were decapitated one day after the behavioral tests (N = 6 for each group). For the western blot data, mice were not subjected to behavioral tests and were decapitated one day after the last exercise regimen in an independent experiment (N = 10 for each group). 

**Figure 2.**
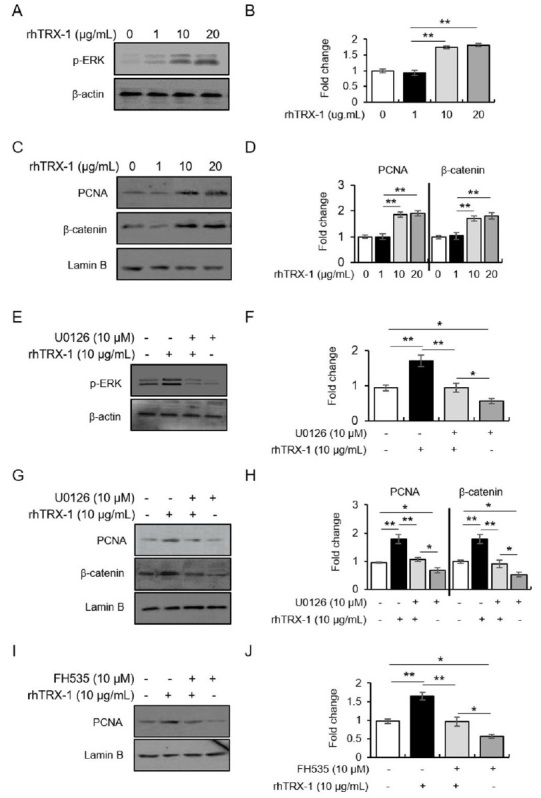
TRX-1-mediated cell proliferation through the ERK1/2/β-catenin/Tcf signaling pathway. (A-B) Representative images of western blot data (A) and quantitative analysis (B) of p-ERK1/2 following rhTRX-1 treatment. (C-D) Representative images of western blot data (C) and quantitative analysis (D) of nuclear PCNA and β-catenin following rhTRX-1 treatment. (E-F) Representative images of western blot data (E) and quantitative analysis (F) of p-ERK1/2 following treatment with rhTRX-1 and U0126. (G-H) Representative images of western blot data (G) and quantitative analysis (H) of nuclear PCNA and β-catenin following treatment with rhTRX-1 and U0126. (I-J) Representative images of western blot data (I) and quantitative analysis (J) of nuclear PCNA following FH535 treatment. Data are presented as the mean ± standard error of the mean. *p < 0.05, **p < 0.01.

In experiment 2 (Fig. 2), we aimed to investigate the indispensable role of ERK/β-catenin in the TRX-1-facilitated proliferation of hippocampal cells. Cytosolic phospho-ERK1/2 (p-ERK1/2), nuclear proliferating cell nuclear antigen (PCNA), and nuclear β-catenin levels were determined after 48 h of incubation with rhTRX-1 containing the cytosolic form of TRX (rhTRX-1, Sigma-Aldrich, St. Louis, MO, USA; 0-20 µg/mL). Also, a mitogen-activated protein kinase/ERK1/2 (MEK) inhibitor, U0126 (Tocris Bioscience, Bristol, UK; 10 µM), or an inhibitor of β-catenin/T cell factor (Tcf)4-mediated transcription, FH535 (Tocris Bioscience, Bristol, UK; 10 µM), was used for pretreatment 30 min before rhTRX-1 treatment (10 µg/mL) and the levels of these agents were measured after incubation. All experiments were performed in four independent replicates. 

**Figure 3.**
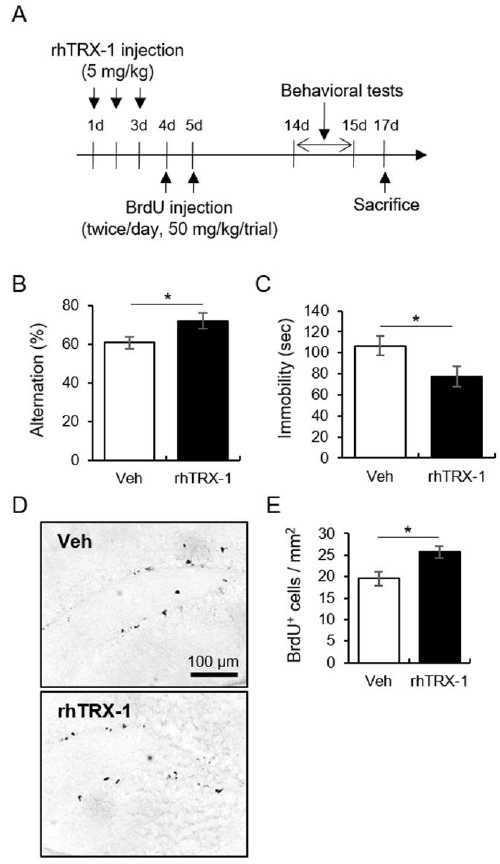
The neurogenic and anti-depressive effects of rhTRX-1. (A) The experimental procedure. (B) Quantitative analysis of alternation in the Y-maze test. (C) Quantitative analysis of immobility in the FST. (D) Representative images of BrdU-labeled cells. (E) Quantitative analysis of BrdU-labeled cells. Data are presented as the mean ± standard error of the mean. *p < 0.05.

In experiment 3 (Fig. 3), to investigate the psychotropic and cell proliferating role of the exogenous rhTRX-1 in mice, rhTRX-1 (5 mg/kg) was intraperitoneally injected for 3 days. Subsequently, BrdU was intraperitoneally injected on the 4th and 5th days after rhTRX-1 treatment. The mice were decapitated 1 day after behavioral tests (N = 10 for each group). 

**Figure 4.**
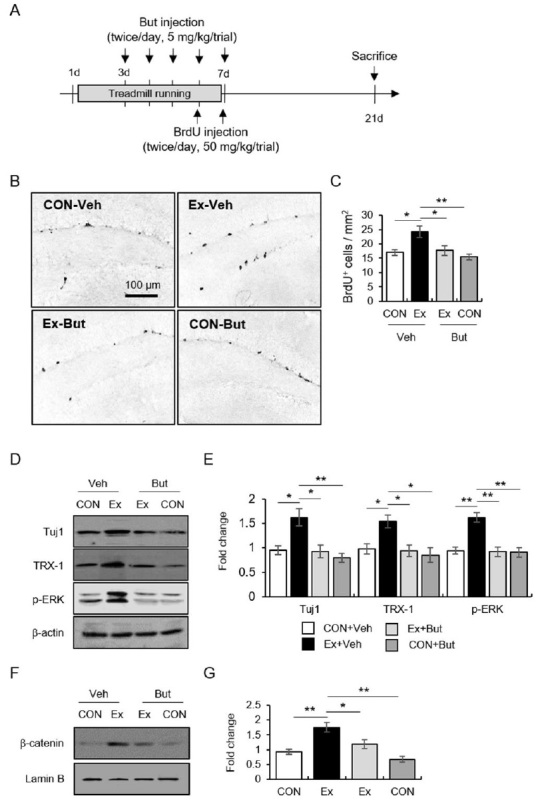
β2-AR function was necessary for the neurogenic effect of exercise via TRX-1-mediated ERK1/2/β-catenin signaling. The effects of But on exercise-induced hippocampal neurogenesis. (A) The experimental procedure. (B) Representative images of BrdU-labeled cells. (C) Quantitative analysis of BrdU-labeled cells. (D-E) Representative images of western blot data (D) and quantitative analysis (E) of Tuj1 and TRX-1 following treatment with But. (F-G) Representative images of western blot data (F) and quantitative analysis (G) of β-catenin following treatment with But. Data are presented as the mean ± standard error of the mean. *p < 0.05.

In experiment 4 (Fig. 4), we verified the regulatory role of exercise-responding β2-AR function in TRX-1-mediated proliferation using a selective β2-AR inhibitor, butoxamine (But, Sigma-Aldrich, St. Louis, MO). Mice were subjected to treadmill running for 7 days. Butoxamine (5 mg/kg, twice per day) was intraperitoneally injected on the 3^rd^ and 7^th^ days of the exercise period, and BrdU was intraperitoneally injected one day after the last exercise regimen. The mice were decapitated 14 days after the last exercise regimen (N = 6 for each group) and western blot analysis was performed (N = 8 for each group). 

### Behavioral assessment

The FST and Y-maze test protocols have been described in a previous publication by our group^24^^,^^25^. In brief, The Y-maze consisted of three equal-sized arms made of white PVC. The arms were 38.5 cm long, 3 cm wide, had 13 cm of height and were oriented at 60° angles between each other (JEUNG DO Bio & Plant Co. LTD, Seoul, Korea). The Y-maze test was performed under moderate lighting conditions (200 lux) with moderately loud background white noise (40 dB). Mice began the trial at the end of one arm and were allowed to explore the Y-maze freely for 8 minutes. The number and sequence of arm visits were recorded manually by an observer. Alternation was defined as consecutive entries in three different arms. The alternation percentage was calculated as following: (number of alternations/total number of arm visits) − 2. For the FST, mice were individually placed in an acrylic cylinder (100 mm diameter × 250 mm height) containing water (24 ± 1°C) to a depth of 17 cm. All mice were exposed to a 15-min pre-test on day 1. The actual test was conducted 24 h later and the experiment was recorded using a video camera. Mice were forced to swim for 6 min and the immobility time was measured after the first min.

### BrdU staining

The mice were subjected to transcardial perfusion, their brains were removed and cryotome sections were collected. Every fifth section from the region between bregma -1.82 mm and -2.18 mm (according to Paxinos and WatsonMouse Brain Atlas) was analyzed. For DNA hydrolysis, tissue sections were washed twice in phosphate-buffered saline, incubated in 2 N HCl for 30 min at 37°C, following neutralization in borate buffer (0.1 M, pH 8.5) for 15 min. Immunohistochemistry (IHC) was performed with specific anti-BrdU primary antibodies (Oxford Biotechnology, Oxford, UK). After incubation with anti-BrdU, sections were incubated with the biotinylated secondary antibody for 1 h at 25 °C and then with avidin-biotin-HRP complex reagent solution (Vector Laboratories, Burlingame, CA, USA). The peroxidase reaction was finally performed using diaminobenzidine tetrahydrochloride (Vector Laboratories). Digital images of IHC staining were captured using a Leica DM750 microscope. Every fifth section was taken from the region between 1.46 to 2.80 mm posterior to bregma. BrdU-positive cells were quantified. 

### Preparation of subcellular fractions and western blot analysis

The DG was extracted from the region between -1.55 mm and -2.79 mm posterior to bregma. The dissected tissue samples that were isolated using tissue punches (0.75 mm diameter) were pooled (2 to 3 samples). Subcellular fraction and western blot were described in a previous publication by our group^26^. Anti-p-ERK1/2 (1:1,000) and anti-TRX-1 (1:1,000) were purchased from Cell Signaling Tech. Inc. (Danvers, MA, USA). β1-AR (1:500) and β2-AR (1:500) were purchased from Abcam (Cambridge, UK). Anti-class III beta-tubulin (anti-Tju1; 1:500), anti-β-catenin (1:500), anti-β-actin, and anti-lamin B were obtained from Sigma-Aldrich (Sigma-Aldrich, St. Louis, MO, USA).

### Primary hippocampal culture

Primary hippocampal cell cultures were prepared from E17 ICR mice. The procedure for hippocampal cell culture has been described in a previous publication by our group^24^. Cultures from day in vitro (DIV) 7 were used for experimental processes. Each in vitro experiment was performed using four independent cultures.

### Statistical analysis

Significant differences between groups were determined using independent t-tests and one-way variance analyses (SPSS for Windows, version 18.0, Chicago, IL, USA). Post-hoc comparisons were made using the least significant difference tests. All values are reported as mean ± standard error of mean (SEM). Values of *p* < 0.05 were considered statistically significant. 

## RESULTS

### Exercise improved the chronic stress-induced depressive phenotype and recovered cell proliferation along with synaptic β-2AR and TRX-1

The mice subjected to continuous 14 days of restraint stress manifested reduced alternations in the Y-maze test and enhanced immobility in the FST, which was reversed by the exercise regimen (Fig. 1A-C; for Y-maze, F_3, 36_ = 7.781, *p* < 0.01; for FST, F_3, 36_ = 10.42, *p* < 0.01). The alternation and immobility of the Ex group were higher and lower than those of the CON group, respectively. In BrdU staining, exercise recovered the BrdU+ cells that were reduced by chronic stress, and exercise alone increased BrdU+ cells compared to the results in control mice (Fig. 1D-E; F_3, 12_ = 11.99, *p* < 0.01). The Tju1 levels were reduced by stress and restored by exercise (Fig. 1F-G; F_3, 12_ = 16.10, *p* < 0.01). Moreover, the exercise regimen restored TRX-1 and synaptic β2-AR protein levels, which had been decreased by chronic stress, and exercise alone enhanced the levels of both proteins relative to those in control mice (Fig. 1F-I; for TRX-1, F_3, 12_ = 25.12, *p* < 0.01; For β2-AR, F_3, 12_ = 28.30, *p* < 0.01; for β1-AR, F_3, 12_ = 1.48, *p* > 0.05).

### Treatment with rhTRX-1 enhanced cell proliferation through the ERK1/2/β-catenin signaling pathway in vitro

To elucidate the facilitating role of TRX-1 in cell proliferation in the hippocampal DG, as well as the contribution of the ERK1/2/β-catenin pathway in TRX-1-induced proliferation, rhTRX-1 (0-20 µg/mL) was used as treatment in hippocampal primary culture with/without pretreatment with a selective MEK inhibitor, U0126 (10 µM). The treatment with rhTRX-1 increased PCNA (a cell proliferation marker), p-ERK1/2, and nuclear β-catenin levels in a dose-dependent manner (Fig. 2A-D; p-ERK1/2, F_3, 12_=57.77, *p* < 0.01; for β-catenin, F_3, 12_=15.47, *p* < 0.01; for PCNA, F_3, 12_=27.23, *p* < 0.01). These increases in protein levels observed in rhTRX-1-treated cultures were reversed by the pretreatment with U0126, and treatment with U0126 alone reduced them in comparison to the basal levels (Fig. 2E-H; p-ERK1/2, F_3, 12_=16.57, *p* < 0.01; for β-catenin, F_3, 12_=22.49, *p* < 0.01; for PCNA, F_3, 12_=24.06, *p* < 0.01). Furthermore, PNCA levels were enhanced by the rhTRX-1 treatment, which was reversed by an inhibitor of β-catenin/Tcf4-mediated transcription, FH535, while treatment with FH535 alone reduced PCNA relative to basal levels (Fig. 2I-J; F_3, 12_ = 24.08, *p* < 0.01). These results indicated that the ERK1/2/β-catenin signaling pathway was required for TRX-1-facilitated proliferation of hippocampal cells.

### Treatment with rhTRX-1 improved depression-like symptoms and cell proliferation in hippocampal DG through the ERK/β-catenin signaling pathway in vivo

We studied the TRX-1-facilitated cell proliferation in the hippocampal DG through ERK1/2/β-catenin signaling and its positive effects on the depressive profile in vivo. The intraperitoneal injection of rhTRX-1 (5 mg/kg) for 3 days enhanced alternation in the Y-maze test and reduced immobility in the FST (Fig. 3A-C; for Y-maze, t_18_ = -2.27, *p* < 0.05; for FST, t_18_ = 2.20, *p* < 0.05). BrdU+ cells in the hippocampal DG were increased by the rhTRX-1 treatment (Fig. 3D-E; t_6_ = -3.01, *p* < 0.05). 

### β2-AR function was necessary for the neurogenic effect of exercise via TRX-1-mediated ERK1/2/β-catenin signaling.

Finally, we explored the controlling role of β2-AR in exercise-induced hippocampal neurogenesis via the TRX-1 pathway using a selective β2-AR antagonist, butoxamine. The intraperitoneal injection of butoxamine (5 mg/kg/trial, twice per day) for 5 consecutive days attenuated the exercise-elicited increase in BrdU+ cells (Fig. 4A-C; F_3, 12_ = 7.27, *p* < 0.01). Also, Tju1 (a differentiating marker) protein levels corresponded well with BrdU staining data (Fig. 4D-E; F_3, 12_ = 8.41, *p* < 0.01). The exercise-induced increases in TRX-1, p-ERK1/2, and nuclear β-catenin levels were reduced by the butoxamine treatment (Fig. 4D-E; for TRX-1, F_3, 12_ = 6.15, *p* < 0.01; for p-ERK1/2, F_3, 12_ = 14.21, *p* < 0.01; for β-catenin, F_3, 12_ = 13.51, *p* < 0.01). These results indicate that the β2-AR-mediated TRX-1 signaling pathway is required for the neurogenic effect of exercise.

## DISCUSSION

The current study demonstrates the psychotropic activity of regular exercise against chronic stress through TRX-1-mediated enhancement of hippocampal neurogenesis. Exercise-responding TRX-1 induction was modulated by β2-AR function in the hippocampal DG. Moreover, we showed that the cell proliferating effect of TRX-1 was controlled by the ERK1/2-β-catenin/Tcf pathway in vitro.

Chronic stress is well known to cause psychiatric illnesses such as depression^1^^-^^2^, whereas exercise has anti-depressive psychotropic effects^16^^-^^17^^,^^19^^-^^21^. The chronic stress paradigm applied in this work caused depressive symptoms, including enhanced despair levels, impaired short-term memory, and decreased hippocampal neurogenesis. On the contrary, the exercise regimen alleviated chronic stress-induced insults. Our observations correspond well with findings from previous studies^18^^,^^24^. Combined with these earlier results, our findings suggest that the experimental paradigm applied in this study was valid for exploring the mechanism of the anti-depressive effects of exercise associated with hippocampal neurogenesis.

In this study, the changes in neurogenic markers (BrdU-labeled cells indicated cell proliferation; Tju1 indicated neuron-specific differentiation) were positively correlated with TRX-1 and β2-AR levels (BrdU vs. TRX-1, r^2^ = 0.75, *p* < 0.01; BrdU vs. β2-AR, r^2^= 0.72, *p* < 0.01). In addition, the outcomes of the exercise only group revealed higher levels of these neurogenic markers, TRX-1, and β2-AR. Alongside its anti-oxidative and anti-apoptotic properties^8^^-^^10^^,^^27^, TRX-1 has been reported to contribute to cell proliferation in tumor, endothelial, adipose tissue-derived mesenchymal stem, and neural precursor cells^11^^-^^12^. A study has shown that hippocampal neurogenesis increased 30 days after treatment with rhTRX-1 in rodents subjected to ischemia and reperfusion^13^. This suggests that TRX-1 might increase the production and survival of newborn hippocampal precursors in late stages following ischemia, even though it exerted an anti-apoptotic effect in early stages. Earlier evidence has suggested a modulatory role of noradrenergic activity in TRX-1 expression. The expression of TRX-1 is modulated by β2-AR, as demonstrated by the use of an adrenergic receptor agonist for its induction and by its inhibition by an adrenergic antagonist^14^^-^^15^. Regarding the hippocampal β2-AR function in relation to stress, a study has shown a decrease in hippocampal β2-AR mRNA and protein expression induced by unpredictable chronic mild stress^28^. In general, exercise increases NA release and noradrenergic activity in various brain regions, including the hippocampus^22^^-^^23^. These previous results support our hypothesis that the neurogenic effect of exercise was at least in part responsible for the TRX-1 induction mediated by β2-AR in hippocampal DG under chronic stress and normal conditions.

The mitogen-activated protein kinase (MAPK) cascade plays a key role in cell proliferation, differentiation, and survival. The ERK1/2 signaling cascade especially contributes to the control of cell proliferation in many cell types, including neural stem or progenitor cells^13^^,^^29^^-^^30^. In terms of adult hippocampal neurogenesis, the β-catenin/Tcf transcriptional activity plays an important role in brain development, including in the self-renewal of neural progenitor cells^31^^-^^33^. We found that the ERK1/2-β-catenin/Tcf pathway was important in TRX-1-induced cell proliferation; in vitro experiments proved that the TRX-1-induced proliferation was attenuated by the inhibition of ERK1/2 or β-catenin/Tcf. Moreover, the sustained inhibition of the ERK1/2-β-catenin/Tcf pathway reduced cell proliferation in this work. These results suggest that this signaling cascade may be a modulatory pathway in the TRX-1-dependent proliferation of hippocampal cells. This interpretation has supporting evidence: ERK1/2 played a crucial role in TRX-1-mediated cell proliferation through the regulation of cell cycle modification with cyclin D1, which is a target of β-catenin/Tcf-dependent transcription in cancer cells^34^^-^^35^. A study observed that TRX-1 overexpression through lentivirus transduction induced the β-catenin/Tcf promoter, which was blocked by U0126 in human adipose tissue-derived mesenchymal stem cells, thus facilitating cell proliferation^12^. A recent study demonstrated that the neurogenic effects of exercise were closely correlated with the GSK3β/β-catenin pathway under chronic stress conditions^18^. The in vitro findings corresponded well with the in vivo results, as observed by the increase in newborn cells in hippocampal DG 14 days after the 3-day treatment with rhTRX-1 in this study. This result implied that TRX-1 facilitated the generation and survival of newborn cells. Furthermore, rhTRX-1 improved the depression profile when compared with the vehicle group, suggesting that TRX-1-induced hippocampal proliferation affects depression-related behavior.

Finally, we confirmed whether the hippocampal neurogenesis facilitated by TRX-1 was directly controlled by β2-AR function during exercise using a selective inhibitor of β2-AR. Since the short-term exercise was sufficient to enhance the generation and survival of BrdU-labeled cells^36^^-^^37^, mice were treated with a β2-AR inhibitor for 5 days during the 7-day exercise period. As expected, the exercise-induced hippocampal neurogenesis was abolished by the β2-AR inhibitor, along with the expression of TRX-1, p-ERK1/2, and nuclear β-catenin. This result provides direct evidence that exercise-induced hippocampal neurogenesis is controlled by the ERK1/2-β-catenin/Tcf signaling pathway through β2-AR function. 

In addition, this neurogenesis may be linked to brain-derived neurotrophic factor (BDNF), as evidenced by the BDNF-induced blockade of photoreceptor degeneration by TRX-1 overexpression and by the activation of TRX-1 by BDNF overexpression^38^^-^^39^. BDNF induction is regulated by 3’5’-cyclic adenosine monophosphate (cAMP) signaling in the hippocampus in learning and memory processes^40^. Accordingly, exercise-induced cAMP signaling may facilitate reciprocal BDNF/TRX-1 interaction in the hippocampal DG area, thereby protecting against depressive symptoms in chronic stress conditions.

Altogether, a probable explanation for our results is that the NA homeostatic disturbance might specifically downregulate β2-adrenergic receptor expression in the hippocampal DG within the context of chronic stress conditions. As opposed to chronic stress, exercise might counter-regulate β2-AR-mediated noradrenergic inputs in the hippocampal DG under noxious conditions. This increase in noradrenergic activity caused by exercise through the activation of the sympathetic nervous system might facilitate hippocampal neurogenesis via TRX-1-dependent ERK1/2-β-catenin/Tcf signaling pathway, thereby improving depressive phenotype under chronic stress. 

This study demonstrated that the neurogenic action of exercise was in part attributed to TRX-1-dependent ERK1/2-β-catenin/Tcf signaling, which modulated NA input into DG. This cascade contributed to the anti-depressive effect of exercise under noxious conditions. Therefore, these findings provide new insight in understanding the psychotropic effects of exercise.
